# The *ISPA*_Int_ Injury Prevention Programme for Youth Competitive Alpine Skiers: A Controlled 12-Month Experimental Study in a Real-World Training Setting

**DOI:** 10.3389/fphys.2022.826212

**Published:** 2022-02-25

**Authors:** Thierry Schoeb, Stefan Fröhlich, Walter O. Frey, Evert Verhagen, Mazda Farshad, Jörg Spörri

**Affiliations:** ^1^Sports Medical Research Group, Department of Orthopaedics, Balgrist University Hospital, University of Zurich, Zürich, Switzerland; ^2^University Centre for Prevention and Sports Medicine, Department of Orthopaedics, Balgrist University Hospital, University of Zurich, Zürich, Switzerland; ^3^Amsterdam Collaboration on Health and Safety in Sports, IOC Research Centre for Prevention of Injury and Protection of Athlete Health, Department of Public and Occupational Health, Amsterdam Movement Sciences, Amsterdam UMC, Amsterdam, Netherlands; ^4^Spine Surgery, Department of Orthopaedics, Balgrist University Hospital, University of Zurich, Zurich, Switzerland; ^5^University Spine Centre, Department of Orthopaedics, Balgrist University Hospital, University of Zurich, Zurich, Switzerland

**Keywords:** athletes, traumatic injuries, overuse injuries, neuromuscular performance, injury prevention, alpine skiing

## Abstract

Evidence-based injury prevention programmes for youth competitive alpine skiers are widely absent. The aims of this controlled 12-month experimental study were to introduce a novel injury prevention programme targeted to the injury patterns of youth skiers, called *ISPA_Int_*, and to compare the differences in injury occurrence between an intervention group (IG) additionally performing the *ISPA_Int_* programme and an independent, historical control group (CG) following their regular training routines. None of the skiers of the CG were part of the IG and vice versa. The study was directly conducted within the real-world youth development structures of skiers competing at the under 16 years (U16) level in Switzerland. Seventy-one skiers (aged 14.4 ± 0.3 years) assigned to the IG were compared to 58 age- and gender-matched controls. The IG was offered the *ISPA_Int_* programme with the recommendation to perform it at least once per week. Skiers’ adherence to this recommendation was surveyed but not enforced. Injuries were recorded using the Oslo Sports Trauma Research Centre Questionnaire. Primary outcomes were the absolute injury rates (number of injuries/100 athletes per season) and epidemiological incidence proportion (number of injured athletes/100 athletes per season). The secondary outcome was the average 2-weekly prevalence of traumatic knee, knee overuse, and lower back overuse injuries. There were lower absolute rates of all traumatic injuries [rate/risk difference, RD: −57.1 (−98.1, −16.0); rate/risk ratio, RR: 0.665 (0.485, 0.884)] and overuse injuries [RD: −35.9 (−71.0, −0.7); RR: 0.699 (0.493, 0.989)] in the IG than in the CG. Likewise, the epidemiological incidence proportion for all overuse injuries was smaller in the IG [RD: −28.4 (−44.8, −12.0); RR: 0.598 (0.435, 0.822)], while the proportion of skiers suffering from traumatic injuries did not significantly differ between the groups. Notably, the IG particularity differed from the CG in the average 2-weekly prevalence of knee trauma, knee overuse, and lower back overuse complaints, three of the major injury-related hot spots in youth skiers. Based on these promising results, the *ISPA_Int_* programme may have great potential to prevent injuries in youth competitive alpine skiers, and the underlying exercises should be considered complementary training content at the U16 level.

## Introduction

The benefits of exercise-based injury prevention programmes have been demonstrated in several competitive sports (Junge et al., 2002; Mandelbaum et al., 2005; Michaelidis and Koumantakis, 2014; Riva et al., 2016; Soomro et al., 2016; Mehl et al., 2018; Petushek et al., 2018; Webster and Hewett, 2018; Huang et al., 2020; Pas et al., 2020). However, to the best of our knowledge, apart from a single study specifically focusing on anterior cruciate ligament (ACL) injuries (Westin et al., 2020), evidence-based injury prevention programmes tailored to the specific injury patterns of U16 competitive alpine skiers are lacking.

Typically, exercise-based injury prevention programmes aim at improving athletes’ neuromuscular and proprioceptive performance (Mandelbaum et al., 2005). Effective prevention, however, should be based on sport- and age-specific programmes that consider the epidemiology, mechanisms, and contextual factors of the injuries of the athletes to be protected (van Mechelen et al., 1992; Sugimoto et al., 2016; Plummer et al., 2019). Accordingly, real-world implementation factors should already be taken into consideration in both the development and evaluation phases of sports injury prevention programmes (Bolling et al., 2018; O’brien et al., 2021).

Regarding injury epidemiology, competitive alpine skiing is known as a sport with relatively high injury rates (Florenes et al., 2009; Westin et al., 2012; Bere et al., 2013a; Hildebrandt and Raschner, 2013; Haaland et al., 2016; Müller et al., 2017; Alhammoud et al., 2020; Fröhlich et al., 2020a,b; Peterhans et al., 2020; Schoeb et al., 2020). Particularly striking is the high number of injuries occurring in youth skiers, namely, the under 16-years (U16) category (Schoeb et al., 2020). During the competitive season, the average 2-weekly prevalence was 12.9% for traumatic injuries and 16.1% for overuse injuries. Traumatic injuries in youth skiers primarily relate to the knee, while overuse injuries most frequently affect the knee and lower back (Schoeb et al., 2020). Certainly, there were other locations of injury, but the aforementioned body parts were by far the most frequently affected (Schoeb et al., 2020).

With respect to injury mechanisms, our current knowledge is limited to the level of elite skiers, while studies on youth skiers are widely lacking (Spörri et al., 2017b). Nevertheless, it is plausible to assume similar injury mechanisms, since, at least in the case of traumatic knee injuries, the injury patterns are comparable (Fröhlich et al., 2020a; Schoeb et al., 2020). Mechanisms of severe knee injuries, such as anterior cruciate ligament (ACL) ruptures, typically include a boot-induced drawer of the tibia relative to the femur (Bere et al., 2011a). In other ACL injury mechanisms, dynamic knee valgus plays a key role (Bere et al., 2013b). This tibial anterior drawer can be effectively counteracted by increased eccentric hamstring strength, while dynamic knee valgus collapse may be antagonised by superior leg axis stability. Moreover, excellent stability of the trunk may prevent skiers from getting “out-of-balance” (often preceding the inciting event of traumatic injuries; Bere et al., 2014).

With regard to knee and back overuse injuries, the inherent movement structures and relative loading patterns of modern skiing techniques can be considered nearly equivalent regardless of the level of competition, since the underlying physics are the same (Howe, 2001). In this connection, superior leg axis stability may prevent the accumulation of excessive nonaxial knee joint loadings (valgus malalignments), which typically occur while skiing (Zorko et al., 2015). Furthermore, excellent abilities to stabilise the trunk can antagonise the combined occurrence of frontal bending, lateral bending, and torsion in vibration-exposed and highly loaded spines, which is typical for skiing (Spörri et al., 2015, 2017a).

Therefore, the aims of this controlled experimental study were 2-fold: (1) to introduce a novel injury prevention programme targeted to the specific injury patterns of youth competitive alpine skiers of the U16 category, hereinafter called *ISPA_Int_* (short for “Injury Screening and Prevention—Alpine Skiing”); and (2) to compare the differences in injury occurrence between an intervention group additionally performing the *ISPA_Int_* programme once a week over a 12-month period in their real-world training setting and age-matched controls following their regular training routines.

## Materials and Methods

### Study Design

**Figure 1:**
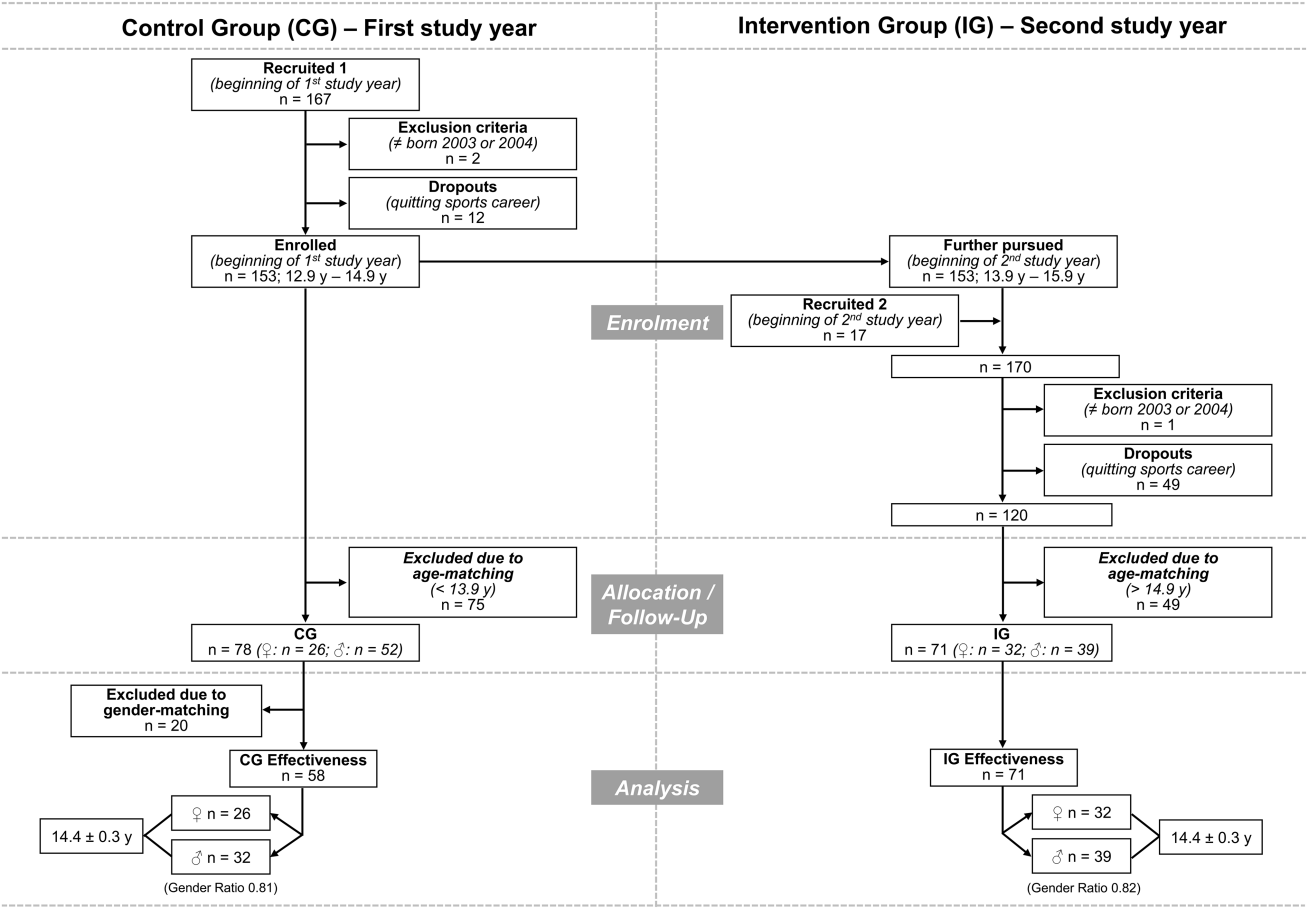
Flow chart of the enrolment, allocation/follow-up, and analysis procedure.

This study (registered at: http://www.clinicaltrials.gov; ID: NCT04021576) was designed as a controlled 12-month experimental study in a real-world training setting of youth competitive alpine skiers. The recruited participants passed through an observation period (first study year; November 2017 to November 2018) and an intervention period (second study year; November 2018 to November 2019). Both study years started with the beginning of the competition season. Based on this pool of data and the specific enrolment/allocation/analysis procedure illustrated in Figure 1 and further described below, participants were allocated to two *independent* groups (i.e., an intervention group—IG vs. a historical control group—CG). To illustrate this in principle, skiers born in 2003 and 2004 typically went through both the observation and intervention periods in 2 consecutive years. The data of the skiers born in 2003 that were collected during the observation period (= first study year) were used for the historical CG, and the data of the skiers born in 2004 that were collected during the intervention period (= second study year) served as the basis for the IG. Accordingly, none of the skiers of the historical CG were part of the IG and vice versa. The exact process of age and gender matching is further described in the corresponding subchapter. The study was designed in such a way that neither the skiers nor the coaches knew about an upcoming intervention period (= second study year). The IG was offered the *ISPA_Int_* programme with the recommendation to perform it once per week. The CG followed their regular training regimes without specific preventative training instructions. Finally, the occurrence of traumatic and overuse injuries was compared between the two groups. The study protocol was approved by the cantonal ethics committee Zurich (KEK-ZH-NR: 2017-01395 and 201801807) and is in conformity with the Helsinki Declaration and national laws. All participants signed an informed consent form. If they were younger than 14 years, their legal guardians signed instead.

### Recruitment

Out of a potential sample of approximately 220 youth competitive alpine skiers of the U16 category in Switzerland, a total of 184 athletes were recruited for a larger study program within the ISPA project (167 at the beginning of the first study year; 17 at the beginning of the second study year; Figure 1). The inclusion criterion was being part of a certified regional performance centre (RLZ/CRP) of Swiss-Ski, i.e., representing the best skiers of their age. Eligible participants were identified based on actual RLZ/CRP team lists and were recruited through official invitation letters and local information events. For the current study, skiers were excluded if they were not born in 2003 or 2004 (i.e., not aged between 12.9 and 14.9 years at the beginning of the first study year). This applied for two participants during the first study year and one during the second study year (Figure 1). A previous study included data of the same initial pool of potential participants (Schoeb et al., 2020); however, the dataset of the historical controls (first study year) in the current study is not identical due to a different purpose and therefore different eligibility criteria. Sampling bias was minimised by the free choice of participation, i.e., each skier could decide whether to participate without negative consequences for the nonparticipating athletes.

### Study Dropouts

Study dropouts were particularly noticeable after the competition season in spring as a direct consequence of not being selected for one of the relevant youth development programmes and were in all cases due to the termination of the participants’ competitive sports careers (12 dropouts during the first study year; 49 dropouts during the second study year; Figure 1). There were no study dropouts due to injury (all injured participants continued their biweekly health reporting until the end of the study year) or simply stopped participation in the study.

### Age- and Gender-Matching

Due to the study design using a historical CG (first year: observation; second year: intervention), systematic age-matching became indispensable (Figure 1). Accordingly, the second years’ data of the younger skiers (born in 2004 and aged 13.9–14.9 years at this time) served for the IG, while age-matched controls relied on the first years’ data of the older skiers (born in 2003 and aged 13.9–14.9 years at that time). Moreover, to directly compare the IG and CG, the corresponding groups were gender-matched. The 20 male participants of the CG who were excluded due to gender-matching were selected by means of a random generator. Finally, 129 youth skiers (aged 14.4 ± 0.3 years), 71 skiers assigned to the IG, and another 58 age- and gender-matched controls (CG), were included in the analysis.

### Randomisation and Blinding

As this study was designed as a 12-month experimental study in a real-world training setting of youth competitive alpine skiers with a historical CG and the participants successively passed through an observation period and an intervention period, randomisation and blinding of the participants was not applicable. The resulting study limitations are further discussed below.

### Intervention

The *ISPA_Int_* programme was developed based on knowledge about alpine skiing-specific injury mechanisms as outlined in the introduction (Bere et al., 2011a,b, 2014; Raschner et al., 2012; Spörri et al., 2015, 2017a,b; Zorko et al., 2015; Jordan et al., 2017; Steenstrup et al., 2018) and was designed as a complementary 20 min home training programme. *ISPA_Int_* was available online with video instructions for illustration purposes and as a hard copy to allow offline usage. *ISPA_Int_* included three main exercise families: (1) eccentric hamstring strength (Dynamic Bridging, Nordic Hamstring Exercise); (2) leg axis stability by strengthening the external hip rotators (Deep Single Leg Pistol Squats); and (3) trunk stability by improving the strength and neuromuscular coordination of the trunk muscles (Dynamic Planking, Deadbug Bridging). For a more detailed description of *ISPA_Int_*, refer to the online Supplementary Material. Again, the intervention group (IG) was offered the *ISPA_Int_* programme with the recommendation to perform it once per week en bloc and exactly as described. The CG followed their regular training regimes without specific preventative training instructions.

### Injury Surveillance

All participants prospectively reported their injuries using the Oslo Sports and Trauma Research Centre (OSTRC)-questionnaire on health problems with 2-week measurement intervals (Clarsen et al., 2014). For data collection and database management, the electronic data capture tool *REDCap®* was used. Every second Monday, an automatic e-mail with a personal questionnaire link was sent to all participants. An automatic reminder followed 2 days later. In case participants did not respond within 3 days, the study team contacted them and their parents personally *via* text message. The questionnaire link remained valid until 7 days after sending.

At the end of each study year, all participants underwent supplementary retrospective medical interviews and clinical examinations, verifying the correctness and completeness of all OSTRC questionnaire-based data entries. During these interviews, all prospective data entries in the online OSTRC questionnaire were discussed and checked together with each participant. Any discrepancy between the online questionnaire and interview data was manually corrected in the database based on the clarifying interview content. Potential recall bias was counteracted by fusing the retrospective information with the prospectively collected data, and in case someone could not remember during the interview, the online entries that were collected prospectively over the year were given priority.

Based on the self-reported classifications in the OSTRC-questionnaires, each documented injury was classified as either *traumatic* or *overuse* injury. A traumatic injury was defined as any physical complaint with a clearly identifiable inciting event; for overuse injuries, such an event was absent. Additionally, injuries were subcategorised as being *substantial* when leading to moderate to severe reduction in training volume or sports performance or to complete inability to participate (i.e., option 3, 4, and 5 in either question 2 or 3 of the OSTRC questionnaire), as defined by Clarsen et al. (2014).

### Outcomes

#### Baseline Characteristics

Each participant underwent a baseline assessment at the beginning of the first and second study years. Anthropometric measures were assessed, including *body height*, sitting height (measuring tape with 0.5 cm intervals), and *body mass* (weighing device with a 0.1 kg scale). To determine each participant’s *maturity offset* (the time before or after maximal growth rate) and subjects’ age at peak height velocity (*APHV*), the noninvasive methodology proposed by Mirwald and Colleagues was used (Mirwald et al., 2002; Malina et al., 2007; Sherar et al., 2007; Müller et al., 2015).

#### Adherence to Intervention

All participants of the IG were asked to perform the *ISPA_Int_* programme at least once per week en bloc and exactly as described. Participants’ adherence to this recommendation was surveyed but not enforced. The IG participants’ adherence to the *ISPA_Int_* programme was assessed by an additional question attached to the OSTRC questionnaire, namely, “please enter the number of training sessions in the last 2 weeks or since the last questionnaire was filled in, in which you have carried out the ISPA prevention programme.” Answers were provided as integers.

#### Response Rates

Response rates to the prospective OSTRC questionnaires and supplementary retrospective interviews were monitored and, with the exception of reminders, were not enforced by negative consequences for nonresponse.

#### Primary Outcome

The primary outcome measure was the incidence of traumatic and overuse injuries, both assigned to an *all* and *substantial* injury category according to their severity. Injury incidence was expressed as the *absolute injury rates* (i.e., number of injuries/100 athletes per season), as well as *epidemiological incidence proportion*, an estimator of the overall injury risk to suffer at least one injury during one season (i.e., the number of injured athletes/100 athletes per season).

#### Secondary Outcome

The secondary outcome measure was the OSTRC questionnaire-based measure *average 2-weekly prevalence* of traumatic knee injuries, knee overuse injuries, and lower back overuse injuries, representing the most typical health issues in alpine skiers (Fröhlich et al., 2020a; Schoeb et al., 2020).

### Sample Size

Under the assumption of a 50% reduction in the U16 alpine skier-specific *absolute rates* of traumatic injuries (132.3 injuries/100 athletes per season) and overuse injuries (112.3 injuries/100 athletes per season) reported previously by Schoeb et al. (2020), i.e., Cohen *d* > 0.575, *α* = 0.05, and 1–*β* = 0.90 and an allocation rate of 71/58 = 1.224, an *a priori* power analysis revealed that a total sample size of at least *n* = 108 skiers (IG *n* = 59; CG *n* = 49) would provide sufficient power for analysing the effect of *ISPA_Int_*.

### Statistical Analysis

Baseline characteristics of the IG and the CG were presented as the *mean ± SD*, and corresponding group differences were analysed by unpaired sample *t* tests (*p* < 0.05). Injury incidence was reported as *absolute injury rates*, as well as *epidemiological incidence proportions* along with their 95% CIs. Differences in injury incidence between the IG and the CG were analysed using the absolute association measures *rate/risk difference* (*RD*), i.e., IG incidence—CG incidence, as well as the relative association measures *rate/risk ratio* (*RR*), i.e., IG incidence/CG incidence, and were reported along with corresponding 95% CIs. Moreover, potential differences were statistically tested based on the Poisson model and *Z*-tests (*z* score > 1.96). Finally, the OSTRC questionnaire-based 2-weekly prevalence of traumatic knee, knee overuse, and lower back overuse injuries (i.e., according to Schoeb et al. (2020), the most typical health issues in youth skiers) was visualised over time, and the IG and CG were compared based on unpaired sample *t* tests (*p* < 0.05).

## Results

### Baseline Characteristics, Adherence to Intervention, and Response Rates

After age- and gender-matching, there were no significant differences in any of the baseline characteristics (Table 1). The IG performed the *ISPA_Int_* programme on average 0.8 ± 0.6 times/week (min: 0.0 times/week; max: 2.4 times/week). Despite being offered the *ISPA_Int_* programme, six of the 71 skiers of the IG did not use the programme at all. The average OSTRC questionnaire response rates for the IG and the CG were 93.0 ± 4.5 and 97.7 ± 3.0, respectively. The participation rate in the supplementary retrospective interviews was 100.0% for both groups.

**Table 1:** Baseline characteristics and intervention adherence.

	Intervention group (*n* = 71)	Control group (*n* = 58)
Age (years)	14.4 ± 0.3	14.4 ± 0.3
Female/male ratio (–)	0.82	0.81
Body mass (kg)	52.5 ± 8.9	52.6 ± 8.6
Body height (cm)	163.4 ± 7.0	163.2 ± 7.2
Maturity offset (y)	0.9 ± 1.0	0.8 ± 1.1
APHV (y)	13.4 ± 1.0	13.6 ± 1.1
*ISPA_Int_* adherence (# sessions per week)	0.8 ± 0.6	–

Baseline data are expressed as the mean ± SD or ratio. Based on an unpaired sample *t*-test, there were no significant differences between the IG and the CG at *p* < 0.05. Tests were backed up by bias-corrected accelerated (BCa) bootstrapping with 10,000 samples. APHV, age at peak height velocity; ISPA_Int_, injury prevention programme tailored to youth skiers.

### IG to CG Differences

#### Primary Outcome Measures

The IG to CG differences with respect to the absolute injury rates are presented in Table 2. Compared to the CG, there were lower rates of all traumatic injuries in the IG [RD: −57.1 (−98.1, −16.0) injuries/100 athletes per season; RR: 0.665 (0.485, 0.884)] and overuse injuries [RD: −35.9 (−71.0, −0.7) injuries/100 athletes per season; RR: 0.699 (0.493, 0.989)]. Moreover, the rate of substantial overuse injuries was lower in the IG than in the CG [RD: −25.6 (−48.5, −2.7) injuries/100 athletes per season; RR: 0.536 (0.309, 0.930)], while the rates of substantial traumatic injuries did not significantly differ between the groups.

**Table 2:** Injury incidence expressed as absolute injury rates (i.e., the number of injuries/100 athletes per season) of traumatic and overuse injuries for the intervention group (IG) and control group (CG).

	Number of injuries		Absolute injury rates (*# injuries/100 athletes per season*)				Rate ratio (RR)	*z*-score
	IG (*n* = 71)	CG (*n* = 58)	IG (*n* = 71)	CG (*n* = 58)	Rate difference (RD)		
All							
Traumatic injuries	77	96	108.5 (82.4, 132.7)	165.5 (132.4, 198.6)	−57.1 (−98.1, −16.0)	0.655 (0.485, 0.884)	2.726
Overuse injuries	59	69	83.1 (61.9, 104.3)	119.0 (90.9, 147.0)	−35.9 (−71.0, −0.7)	0.699 (0.493, 0.989)	1.998
Substantial							
Traumatic injuries	58	61	81.7 (60.7, 102.7)	105.2 (78.8, 131.6)	−23.5 (−57.2, 10.3)	0.777 (0.542, 1.113)	1.364
Overuse injuries	21	32	29.6 (16.9, 42.2)	55.2 (36.1, 74.3)	−25.6 (−48.5, −2.7)	0.536 (0.309, 0.930)	2.188

Incidence data are expressed as absolute injury rates with 95% CIs in parentheses. Rate differences (RDs) and rate ratios (RRs) are presented as association measures representing the absolute and relative rate reductions, respectively. Level of significance based on the Poisson model and *Z*-tests for comparing absolute injury rates between groups: *z* score > 1.96.

The IG to CG differences with respect to the epidemiological incidence proportion are summarised in Table 3. The risk of suffering at least one overuse injury of any severity during the season was significantly lower in the IG than in the CG [RD: −28.4 (−44.8, −12.0) injured athletes/100 athletes per season; RR: 0.598 (0.435, 0.822)]. The same applies to substantial overuse injuries [RD: −16.5 (−31.9, −1.0) injured athletes/100 athletes per season; RR: 0.545 (0.305, 0.973)], while the proportion of athletes suffering from traumatic injuries (both all and substantial) did not significantly differ between the groups.

**Table 3:** Injury incidence expressed as the epidemiological incidence proportion, an estimator of the overall injury risk to suffer at least one injury during one season (i.e., the number of injured athletes/100 athletes per season), of traumatic and overuse injuries for the intervention group and control group.

	Number of injured athletes		Epidemiological incidence proportion (*# injured athletes/100 athletes per season*)			Risk ratio (RR)	*z*-score
	IG (*n* = 71)	CG (*n* = 58)	IG (*n* = 71)	CG (*n* = 58)	Risk difference (RD)		
All
Traumatic Injuries	48	39	67.6 (66.3, 68.9)	67.2 (65.7, 68.8)	0.4 (−15.9, 16.6)	1.005 (0.790, 1.280)	−0.044
Overuse Injuries	30	41	42.3 (40.9, 43.6)	70.7 (69.2, 72.2)	−28.4 (−44.8, −12.0)	0.598 (0.435, 0.822)	3.230
Substantial							
Traumatic Injuries	39	33	54.9 (53.6, 56.3)	56.9 (55.2, 58.6)	−2.0 (−19.2, 15.2)	0.965 (0.710, 1.313)	0.224
Overuse Injuries	14	21	19.7 (18.6, 20.8)	36.2 (34.6, 37.8)	−16.5 (−31.9, −1.0)	0.545 (0.305, 0.973)	2.095

Incidence data are expressed as epidemiological incidence proportions with 95% CIs in parentheses. Risk differences (RDs) and risk ratios (RRs) are presented as association measures representing the absolute and relative risk reductions, respectively. Level of significance based on the Poisson model and *Z*-tests for comparing injury incidences between groups: *z* score > 1.96.

#### Secondary Outcome Measures

**Figure 2:**
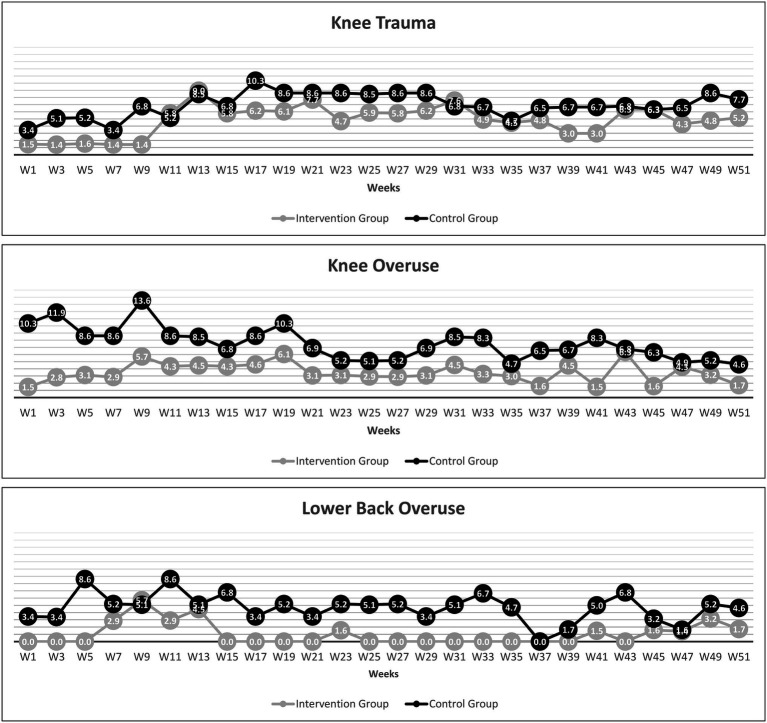
Time course of the average 2-weekly prevalence for knee trauma, knee overuse, and lower back overuse complaints (any severity) over the 12-month observation period.

Figure 2 illustrates the average 2-weekly prevalence of the most typical health issues in alpine skiers over time.

The average 2-weekly prevalence of knee trauma, knee overuse, and lower back overuse injuries of any severity over the entire observation period was significantly lower in the IG than in the CG (Table 4).

**Table 4:** Average 2-weekly prevalence of knee trauma, knee overuse, and lower back overuse complaints (any severity), representing the most frequent health issues in alpine skiers.

Average 2-weekly prevalence (%)			
Injury type			*p* value	Cohen *d*	Power
	Intervention Group (*n* = 71)	Control Group (*n* = 58)			
Knee trauma	4.8 (4.0, 5.6)	6.9 (6.3, 7.6)	<0.001	−0.974	1.000
Knee overuse	3.5 (3.0, 4.0)	7.5 (6.7, 8.4)	<0.001	−1.466	1.000
Lower back overuse	1.0 (0.4, 1.6)	4.7 (3.9, 5.4)	<0.001	−1.427	1.000

Prevalence data are expressed as the mean percentage values with 95% CIs in parentheses. Level of significance based on unpaired sample *t* tests and backed up by bias-corrected accelerated (BCa) bootstrapping with 10,000 samples: *p* < 0.05. Substantial health problems were defined in accordance with Clarsen et al. (2014).

## Discussion

The most important finding of the study was the significantly lower absolute rates of all traumatic injuries (−33.5%) and overuse injuries (−30.1%) in the IG than in the CG. Similarly, the epidemiological incidence proportion for all overuse injuries was 40.2% lower in the IG, while the number of skiers who suffered at least one traumatic injury per season did not significantly differ between the groups. It was also found that the average 2-weekly prevalence of knee trauma, knee overuse, and lower back overuse complaints (any severity) was lower in the IG than in the CG.

### The *ISPA_Int_* Programme—Promising Results Toward Effective Injury Prevention in Youth Competitive Alpine Skiers

As shown in this controlled experimental study, the youth skiers performing the *ISPA_Int_* programme on average 0.8 ± 0.6 times/week over a 12-month period in addition to their regular training regimens showed lower absolute rates of traumatic and overuse injuries. This equally applies to both the *all* and *substantial* injury categories, with the exception of substantial traumatic injuries, which may be difficult to prevent given the high speeds and forces involved in skiing (Gilgien et al., 2014). Likewise, the proportion of youth skiers suffering from at least one overuse injury was lower in the IG. This proportion, however, was not significantly smaller for traumatic injuries, which might be explained by the high number of traumatic injuries occurring in youth skiers (more than 1.3 traumatic injuries per athlete per season; Schoeb et al., 2020). Accordingly, despite the smaller total number, not every prevented injury means one affected athlete less.

The absolute rates of all traumatic and overuse injuries differed between the IG and CG by 33.5 and −30.1%, respectively. Similar effect magnitude ranges have been reported in previous randomised controlled trials assessing the efficacy of neuromuscular and proprioceptive injury prevention programmes in different sports (Soomro et al., 2016; Petushek et al., 2018; Webster and Hewett, 2018; Huang et al., 2020). Knowing that the effect of an intervention is presumed to decrease as testing moves from efficacy to effectiveness to dissemination and implementation research stages, the results of the current controlled experimental study that was conducted under unenforced real-world implementation conditions can be considered promising. Moreover, in addition to the recent study by Westin et al. (2020) who reported a 45% reduction in the ACL injury incidence rate in U18 skiers, this is the first exercise-based prevention study focusing on younger skiers and different types of injuries.

Notably, the IG differed from the CG in the average 2-weekly prevalence of traumatic knee injuries, knee overuse injuries, and lower back overuse injuries, three of the major injury-related hot spots in youth skiers (Westin et al., 2012; Müller et al., 2017; Fröhlich et al., 2020a,b; Peterhans et al., 2020; Schoeb et al., 2020). This may confirm that the aetiology-based derivation of the *ISPA_Int_* programme described in the introduction is sound and that the programme may be effective in counteracting the typical sport-specific injury mechanisms and adverse loading patterns (Bere et al., 2011a,b, 2014; Spörri et al., 2015, 2017a; Zorko et al., 2015; Steenstrup et al., 2018). However, to conclusively confirm our controlled experimental observations, further randomised controlled trials are required.

### Potential Real-World Implementation Pitfalls and Countermeasures

Implementing a sports injury prevention programme in a real-world setting is challenging (Donaldson et al., 2017), which is why incorporating the context of the implementation setting is of great importance (Durlak and DuPre, 2008). Accordingly, we have set on a simple complementary training programme that can be conducted anytime and anywhere within 20 min. Moreover, it was matched to the athletic long-term development strategy of Swiss-Ski.

Additionally, how an injury prevention programme is delivered and supported is known to play an important role in its effect (Durlak and DuPre, 2008). Consequently, high-quality implementation should build upon a partnership between programme developers (researchers) and programme implementers (gatekeepers and end users; Donaldson et al., 2017). In our study, the skiers and their direct personal environment (e.g., coaches and parents) were actively involved in programme development and implementation, an approach that is also strongly recommended for later nonstudy-related scaling-ups of *ISPA_Int_*.

### Why Our Prevention Efforts Should Focus on Youth Competitive Alpine Skiers

Overall, the high injury rates and risks observed in this study further highlight the substantial burden of injury in skiers of the U16 category (Schoeb et al., 2020). In fact, it is known that during phases of accelerated growth around APHV, neuromuscular adaptation processes are decelerated (Backous et al., 1988), making youth skiers especially prone to injuries (Fröhlich et al., 2020b; Peterhans et al., 2020; Schoeb et al., 2020). Moreover, in view of an up to 15 times higher rate for a second injury after ACL reconstruction in adolescent athletes (Paterno et al., 2012), as well as a 1–6-fold higher risk of osteoarthritis development after knee injury (Poulsen et al., 2019), preventing a skiers’ first severe injuries at the youth level must be a priority.

Additionally, our finding of fewer injuries occurring in the IG, just additionally performing a simple 20′ home-based prevention programme, underlines the great preventative potential in this specific target group. Unlike many other youth sports, the training of U16 skiers is still semi-professional, and not all skiers have access to health management experts such as team physicians or physiotherapists.

### Methodological Considerations

A first limitation of the study might be seen in the lack of randomisation due to the use of a historical control group. The well-known confounding factors of calendar age/biological maturity and sex (Schoeb et al., 2020) were addressed by systematic age- and gender-matching. The potential confounding effect of participants becoming more aware of the injury problem throughout the study was counteracted by not providing participants with direct feedback on interim findings (such as the general injury risks observed in the entire study population) or recommendations on possible countermeasures during the study year. The only exception was the *ISPA_Int_* programme that was offered to the IG at the beginning of the second study year. Nevertheless, in a complex and multifactorial system of injury causation, some risk of bias from unknown confounding factors may remain, which certainly limits the conclusions that can be drawn regarding cause and effect. However, as already stated above, the study was conducted within the real-world youth development structures of the Swiss national ski federation (Swiss-Ski). The potential to intervene in such an existing training structure, as well as the pool of potential study participants (i.e., youth competitive alpine skiers of the U16 category in Switzerland), was therefore certainly limited. Under such circumstances, the reasons for choosing a historical control group were twofold: (1) a randomised controlled trial, i.e., instructing certain randomly assigned athletes to perform a specific prevention programme while controlling them with their direct teammates, would have introduced a substantial risk for crossover effects; and (2) a cluster randomised trial would not have been a feasible alternative, as such an approach is known to require a larger number of participants to obtain equivalent statistical power (Campbell et al., 2004). Thus, both alternatives would have severely undermined the validity of the current study.

A second limitation may be seen in the fact that the programme was only conducted once a week for approximately 20 min. However, if one transfers the theoretical effects of efficacy studies to the real world, as was done in this study, a prevention programme certainly suffers from a so-called “voltage drop” (Chambers et al., 2013), i.e., a decreased effect, and a once-a-week implementation may be much more realistic than the 2–3 sessions usually investigated in standard randomised controlled trials. Thus, the reduction in injury rate of approximately one third observed in this study can at least be related to a realistic frequency of intervention and an actual compliance that can be realistically achieved.

A third limitation of the study is that a simple home-based injury prevention programme does not allow any quantity and quality control by an experienced coach or physiotherapist. This has been counteracted by defining a programme that is self-explanatory and by providing detailed exercise descriptions and video tutorials that highlight the key points for exercise execution. Nevertheless, at the youth level of competitive alpine skiing, a simple home-based training programme is likely to be better suited to the real-world training structures of youth competitive alpine skiers than multimodal 1:1 training with a professional health expert or personal trainer.

A fourth limitation of the study is the self-reported type of data, which relies on the correctness and quality of the answers provided. This may imply the risk of suffering from recall and/or reporting bias. Recall bias was counteracted by prospective data collection with 2-week intervals (OSTRC questionnaire), which also allowed the recall of less severe injuries. Reporting bias was antagonised by supplementary retrospective interviews at the end of each study year aiming to verify the correctness and completeness of the self-reported data. If a relevant injury had not been reported due to the lack of a biweekly questionnaire response, the missing injury would have been discovered and entered into the database *via* the supplementary interview. Accordingly, despite slightly lower OSTRC questionnaire response rates in the IG than in the CG, the merged data quality can be considered equivalent.

Finally, regarding overuse injuries to the knee, there was an apparent difference in the average 2-weekly prevalence between the IG and the CG at baseline, without the exact reasons being known. Accordingly, the mean 12-month difference between the IG and CG in terms of overuse injuries to the knee must be interpreted with some caution.

## Conclusion

Based on the promising results of this controlled experimental study, the *ISPA_Int_* programme (a sports injury prevention programme including exercises for eccentric hamstring strength, leg axis stability, and trunk stability) may have great potential to prevent traumatic and overuse injuries in youth competitive alpine skiers, and the underlying exercises should be considered fundamental complementary training content at the U16 level.

## Data Availability Statement

The datasets presented in this article are not readily available because their access is restricted to protect the interests of the project partner Swiss-Ski and their athletes. Requests to access the datasets should be directed to joerg.spoerri@balgrist.ch.

## Ethics Statement

The studies involving human participants were reviewed and approved by Cantonal ethics committee Zurich (KEK-ZH-NR: 2017-01395 and 2018-01807). Written informed consent to participate in this study was provided by the participants’ legal guardian/next of kin.

## Author Contributions

JS and WF conceptualised and designed the study. JS recruited the participants and organised the data collection. TS, SF, and JS collected the data and processed the data and performed the statistical analysis. EV advised on the evaluation methodology. TS, SF, WF, EV, MF, and JS substantially contributed to the interpretation of data. TS and JS drafted the current manuscript. All authors contributed to the article and approved the submitted version.

## Funding

This study was generously supported by the Balgrist Foundation, Swiss-Ski, the “Stiftung Passion Schneesport,” and the “Stiftung zur Förderung des alpinen Skisportes in der Schweiz (SFSS).”

## Conflict of Interest

The authors declare that the research was conducted in the absence of any commercial or financial relationships that could be construed as a potential conflict of interest.

## Publisher’s Note

All claims expressed in this article are solely those of the authors and do not necessarily represent those of their affiliated organizations, or those of the publisher, the editors and the reviewers. Any product that may be evaluated in this article, or claim that may be made by its manufacturer, is not guaranteed or endorsed by the publisher.

## Supplementary Material

The Supplementary Material for this article can be found online at: https://www.frontiersin.org/articles/10.3389/fphys.2022. 826212/full#supplementary-material

File A *ISPA_Int_* programme in English.

File B *ISPA_Int_* programme in German.

File C *ISPA_Int_* programme in French.

File D *ISPA_Int_* programme in Italian.

Video 1 Dynamic bridging.

Video 2 Nordic hamstring exercise.

Video 3 Single leg squat (Floor).

Video 4 Single leg squat (Box).

Video 5 Dynamic planking.

Video 6 Deadbug bridging.
