# Supplementary material for: The ISPAInt Injury Prevention Programme for Youth Competitive Alpine Skiers: A Controlled 12-Month Experimental Study in a Real-World Training Setting
Source: Front Physiol. 2022 Feb 25;13:826212. doi: 10.3389/fphys.2022.826212 (PMC8929391; doi:10.3389/fphys.2022.826212)
Supplement: File A — ISPAInt programme in English. [file Data_Sheet_1.PDF]

Balgrist
University Hospital

swissski

ISPA Prevention Programme

Dynamic Bridging | Nordic Hamstring Exercise | Single Leg Squat
Dynamic Planking | Deadbug Bridging

	Static / Dynamic	Exercise	Instruction	Sets
**Hamstrings 1**	Dynamic	**Dynamic Bridging**<br>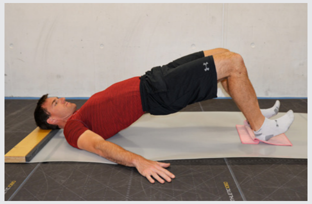<br>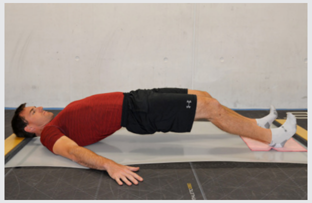	**Starting position:**<br>• Supine position; head resting on the ground; barefoot<br>• Arms extended 45° with both palms facing down<br>• Knees flexed and hip width apart<br>• Heels placed on a sliding pad (towel, carpet, slide, . . .)<br>• Pelvis is raised to the extent where shoulder, hip and knee form a straight line (from a lateral point of view)<br>• Flexed knees (90°)<br>• Core muscles are contracted to maintain the natural position of the lumbar spine (lordosis) at all times<br><br>**Exercise execution:**<br>• Feet flexed; heels are pushed away from body out of the starting position (2 sec)<br>• When body is fully extended, the position is held (1 sec)<br>• Retract legs until starting position is regained (2 sec)<br>• When starting position is regained, immediately start next repetition<br><br>**Focus:**<br>Throughout the entire exercise:<br>• Shoulder, hip and knee (from a lateral point of view) form a straight line<br>• The pelvis is elevated throughout the entire exercise	Repetitions per Set: 8–12<br>Sets: 2<br>Rest between Sets: 1 Min<br>Timing: see exercise execution
**Hamstrings 2**	Dynamic	**Nordic Hamstring Exercise**<br>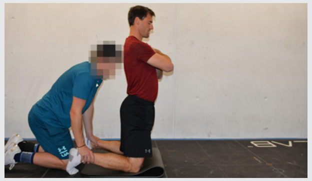<br>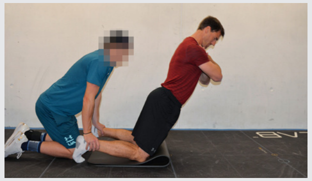<br>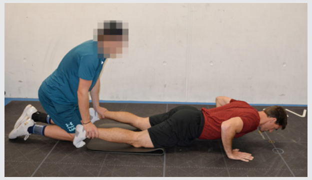	**Starting position:**<br>• In kneeling positioning with propped up feet (progression: additional underlay, e.g. black roll under both ankles), barefoot<br>• Legs held in place at the ankles by a partner<br>• Shoulder, hip and knee form a straight line (from a lateral point of view)<br>• Arms crossed over the chest<br><br>**Exercise execution:**<br>• Continuously lean forward from the starting position (3 sec)<br>• Both legs try to maximally resist the forward movement<br>• Execute movement until position can no longer be held. Absorb a fall with both arms and return to starting position (Ideally under maintained hip extension)<br><br>**Focus**:<br>• Shoulder, hip and knee form a straight line (from a lateral point of view) throughout the entire exercise	Repetitions per Set: 3–6<br>Sets: 2<br>Rest between Sets: 1 Min<br>Timing: see exercise execution<br>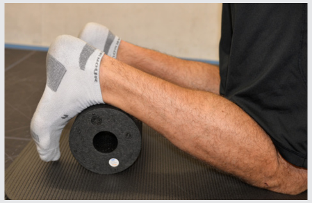

	Static / Dynamic	Exercise	Instruction	Sets
**Leg axis 1**	Dynamic	**Single Leg Squat (right)**<br>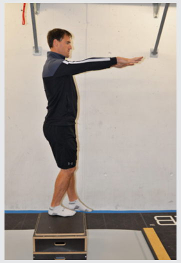 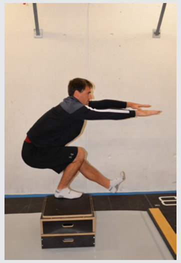	**Starting position:**<br>• Right sided one-leg stand on a box (progression: one-leg stand on ground), barefoot<br>• Arms extended to the front at shoulder height parallel to ground<br>• Left leg extended forward<br>**Exercise execution:**<br>• The pivot leg is bent until thigh is parallel to the ground (2 sec)<br>• Position is held (1 sec)<br>• Pivot leg is dynamically extended until starting position is regained<br>**Focus:**<br>• Buttock muscles are **consciously** contracted<br>• Hip and leg axis are kept stable throughout the entire exercise	Repetitions per Set: 6–8<br>Sets: 2<br>Rest between Sets: 1 Min<br>Timing: see exercise execution<br>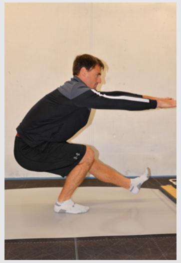
**Leg axis 2**	Dynamic	**Single Leg Squat (left)**<br>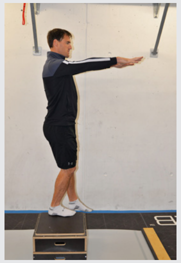 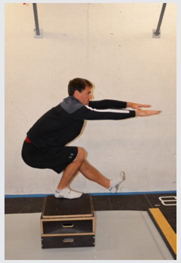	**Starting position:**<br>• Left sided one-leg stand on a box (progression: one-leg stand on ground), barefoot<br>• Arms extended to the front at shoulder height parallel to ground<br>• Right leg extended forward<br>**Exercise execution:**<br>• The pivot leg is bent until thigh is parallel to ground. (2 sec)<br>• Position is held (1 sec)<br>• Pivot leg is dynamically extended until starting position is regained<br>**Focus:**<br>• Buttock muscles are **consciously** contracted<br>• Hip and leg axis are kept stable throughout the entire exercise	Repetitions per Set: 6–8<br>Sets: 2<br>Rest between Sets: 1 Min<br>Timing: see exercise execution<br>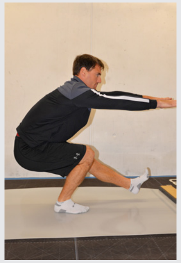

	Static/ Dynamic	Exercise	Instruction	Sets
**Core 1**	Static	**Dynamic Planking**<br>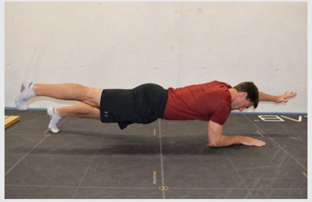	**Starting position:**<br>• Prone Planking position (weight on lower arms and tiptoes), barefoot<br>• Elbow directly placed under shoulder, lower arms parallel to the body's longitudinal axis<br>• Head, shoulder, hip and knee form a straight line (from a lateral point of view)<br>• Core and buttock muscles contracted, slight tension in the shoulder blade area<br>**Exercise execution:**<br>• Simultaneous elevation of arm and leg opposite each other<br>• Hold position (2 sec)<br>**Focus:**<br>• Core and hips are kept stable (head, shoulder, hip and knee form a straight line) throughout the entire exercise<br>• Natural positioning of lumbar spine (lordosis) is maintained throughout the entire exercise	Repetitions per Set: 20–30<br>Sets: 2<br>Rest between Sets: 1 Min<br>Timing: see exercise execution
**Core 2**	Static	**Deadbug Bridging**<br>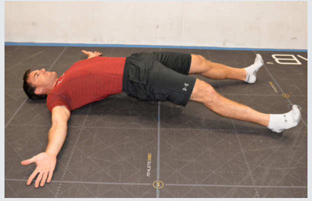<br>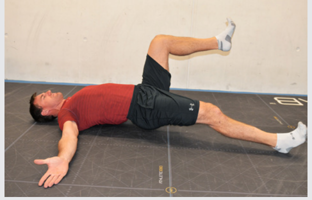	**Starting position:**<br>• Supine; arms 90° extended, palms facing up; barefoot<br>• Legs extended (heels and elbows with same distance from midline of body), tip of toes are hyperextended<br>• Metaphor: Pull belly button towards chin<br>• Tensions is generated in core muscles<br>**Exercise execution:**<br>• Pelvis is slightly elevated (5–10 cm) and maintained in this position for the whole exercise<br>• The knees are alternatingly pulled toward the chest until the thigh is vertical to the ground (2 sec)<br>• Hold position (3 sec)<br>• The leg is slowly lowered to a complete foot rest<br>**Focus:**<br>• Core and hip muscle are kept stable throughout the entire exercise<br>• Maintain the starting position in the spine («pull belly button towards chin») throughout the entire exercise	Repetitions per Set: 4–6<br>Sets: 2<br>Rest between Sets: 30 sec<br>Timing: see exercise execution<br>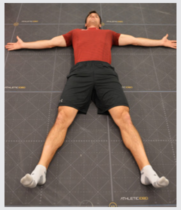

Video-Instructions:
www.swiss-ski.ch/off-snow
