# Supplementary material for: The ISPAInt Injury Prevention Programme for Youth Competitive Alpine Skiers: A Controlled 12-Month Experimental Study in a Real-World Training Setting
Source: Front Physiol. 2022 Feb 25;13:826212. doi: 10.3389/fphys.2022.826212 (PMC8929391; doi:10.3389/fphys.2022.826212)
Supplement: File B — ISPAInt programme in German. [file Data_Sheet_2.PDF]

Balgrist
Universitätsklinik

swissski

ISPA Präventionsprogramm

Dynamic Bridging | Nordic Hamstring Exercise | Single Leg Squat
Dynamic Planking | Deadbug Bridging

	Statisch/ Dynamisch	Übung	Anweisung	Menge
**Hamstrings 1**	Dynamisch	**Dynamic Bridging**<br>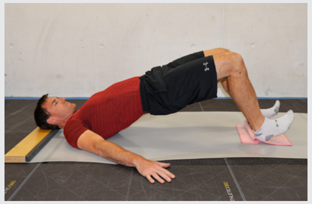<br>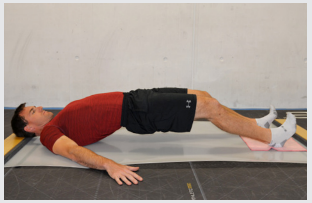	**Ausgangsposition:**<br>• Auf Rücken liegend; Kopf hat Bodenkontakt; barfuss<br>• Arme in 45° vom Körper abgespreizt; Handflächen nach unten<br>• Beine angewinkelt in Hüftbreiter Position<br>• Fersen auf rutschender Unterlage (Tuch, Teppich, Slide, …)<br>• Becken anheben bis Schulter, Hüfte und Knie (von der Seite betrachtet) eine Linie bilden<br>• Unterschenkel in 90° zum Oberschenkel ausrichten<br>• Unter Beibehalten der natürlichen Position der Lendenwirbelsäule (Lordose), Rumpfmuskulatur anspannen<br><br>**Übungsausführung:**<br>• Füsse anwinkeln; Fersen aus Ausgangsposition vom Körper wegschieben (2 Sek)<br>• Sobald komplette Körperstreckung erreicht ist, Position halten (1 Sek)<br>• Beine anziehen bis wieder in Ausgangsposition (2 Sek)<br>• Sobald Ausgangsposition erreicht ist, unmittelbarer Richtungswechsel und Fersen erneut vom Körper wegschieben<br><br>**Fokus:**<br>Während der gesamten Übung:<br>• Schulter-, Hüfte- und Knie bilden (von der Seite brachtet) eine Linie<br>• Kein Bodenkontakt des Gesässes	Wiederholungen pro Serie: 8–12<br>Serien: 2<br>Serienpause: 1 Min<br>Timing: siehe Übungsausführung
**Hamstrings 2**	Dynamisch	**Nordic Hamstring Exercise**<br>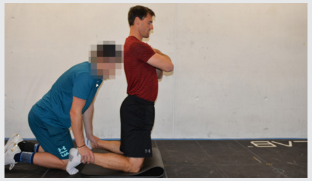<br>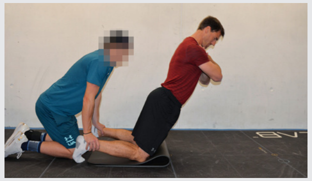<br>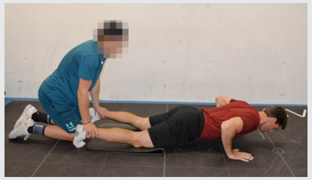	**Ausgangsposition:**<br>• In kniender Position auf Boden mit aufgestützten Füssen (Steigerung: zusätzliche Unterlage, z. B. Blackroll unter Fussgelenke); barfuss<br>• Beine an den Knöcheln durch Partner fixiert<br>• Schulter, Hüfte und Knie bilden (von der Seite betrachtet) eine Linie<br>• Arme vor der Brust überkreuzt<br><br>**Übungsausführung:**<br>• Den Körper aus Ausgangsposition kontinuierlich nach vorne neigen (3 Sek)<br>• Mit beiden Beinen die Bewegung maximal bremsen<br>• Bewegung fortführen bis die Position nicht mehr gehalten werden kann, mit Armen abfedern und in Ausgangsposition zurückkehren (im Idealfall: Hüftstreckung beibehalten)<br><br>**Fokus**:<br>• Schulter, Hüfte und Knie bilden während gesamter Übung eine Linie	Wiederholungen pro Serie: 3–6<br>Serien: 2<br>Serienpause: 1 Min<br>Timing: siehe Übungsausführung<br>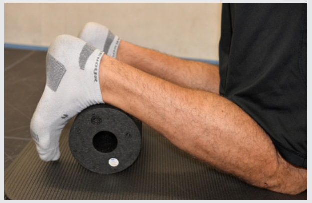

ISPA Präventionsprogramm
entwickelt am Universitären Zentrum für Prävention und Sportmedizin der Universitätsklinik Balgrist

in Kooperation mit
swissski

	Statisch/ Dynamisch	Übung	Anweisung	Menge
**Beinachse 1**	Dynamisch	**Single Leg Squat (rechts)**<br>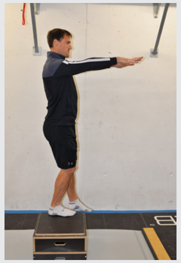 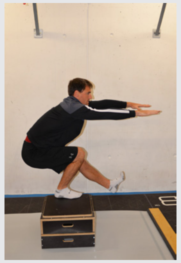	**Ausgangsposition:**<br>• Einbeinstand rechts auf Box (Steigerung: Boden); barfuss<br>• Arme auf Schulterhöhe parallel zum Boden nach vorne strecken<br>• Bein gestreckt in Vorhalteposition<br>**Übungsausführung:**<br>• Standbein beugen bis Oberseite des Oberschenkels parallel zum Boden ist (2 Sek)<br>• Position halten (1 Sek)<br>• Standbein dynamisch strecken bis Ausgangsposition wieder erreicht ist<br>**Fokus:**<br>• Gesässmuskulatur **bewusst** anspannen<br>• Hüftachse und Beinachse bleiben stabil	Wiederholungen pro Serie: 6–8<br>Serien: 2<br>Serienpause: 1 Min<br>Timing: siehe Übungsausführung<br>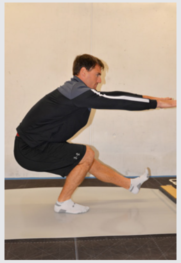
**Beinachse 2**	Dynamisch	**Single Leg Squat (links)**<br>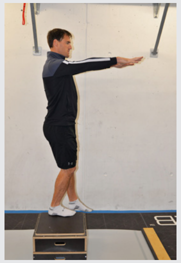 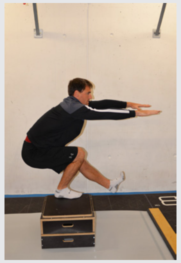	**Ausgangsposition:**<br>• Einbeinstand links auf Box (Steigerung: Boden); barfuss<br>• Arme auf Schulterhöhe parallel zum Boden nach vorne strecken<br>• Bein gestreckt in Vorhalteposition<br>**Übungsausführung:**<br>• Standbein beugen bis Oberseite des Oberschenkels parallel zum Boden ist (2 Sek)<br>• Position halten (1 Sek)<br>• Standbein dynamisch strecken bis Ausgangsposition wieder erreicht ist<br>**Fokus:**<br>• Gesässmuskulatur **bewusst** anspannen<br>• Hüftachse und Beinachse bleiben stabil	Wiederholungen pro Serie: 6–8<br>Serien: 2<br>Serienpause: 1 Min<br>Timing: siehe Übungsausführung<br>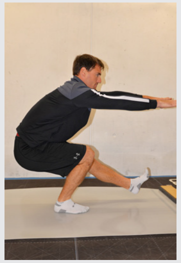

	Statisch/ Dynamisch	Übung	Anweisung	Menge
**Rumpf 1**	Statisch	**Dynamic Planking**<br>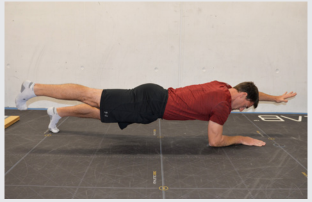	**Ausgangsposition:**<br>• In Bauchlage Stützposition (auf Unterarmen und Zehenspitzen) einnehmen; barfuss<br>• Ellbogen direkt unter der Schulter; Unterarme parallel zur Körperlängsachse ausgerichtet<br>• Kopf, Schulter, Hüfte und Knie bilden (von der Seite betrachtet) eine Linie<br>• Rumpf- und Gesässmuskulatur sind angespannt; leichte Spannung im Bereich der Schulterblätter<br>**Übungsausführung:**<br>• Gegengleiches Anheben von gestreckten Armen & Beinen<br>• Position jeweils 2 Sek halten<br>**Fokus:**<br>• Rumpf und Hüfte bleiben stabil (Kopf, Schulter, Hüfte und Knie bilden eine Linie)<br>• Natürliche Position der Lendenwirbelsäule (Lordose) wird beibehalten	Wiederholungen pro Serie: 20–30<br>Serien: 2<br>Serienpause: 1 Min<br>Timing: siehe Übungsausführung
**Rumpf 2**	Statisch	**Deadbug Bridging**<br>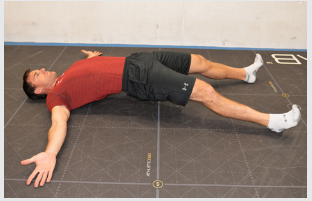<br>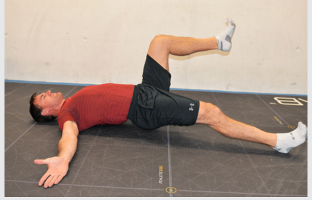	**Ausgangsposition:**<br>• Auf Rücken liegend; Arme in 90° zum Körper; Handflächen nach oben; barfuss<br>• Beine ausgestreckt und abgespreizt (Fersen und Ellbogen ca. gleich weit von der Körpermitte entfernt), Fussspitzen angezogen<br>• Metapher: «Bauchnabel Richtung Kinn ziehen»<br>• Spannung im Rumpf aufbauen<br>**Übungsausführung:**<br>• Becken leicht anheben, und im Abstand von einer Faust über dem Boden halten<br>• Knie wechselseitig Richtung Brust ziehen bis die Oberschenkel senkrecht zum Boden sind (2 Sek)<br>• Position halten (3 Sek)<br>• Bein langsam senken (2 Sek) und Fuss kontrolliert wieder absetzen<br>**Fokus:**<br>• Rumpf und Hüfte bleiben stabil<br>• Ausgangsposition der Wirbelsäule beibehalten («Bauchnabel Richtung Kinn ziehen»)	Wiederholungen pro Serie: 4–6<br>Serien: 2<br>Serienpause: 30 Sek<br>Timing: siehe Übungsausführung<br>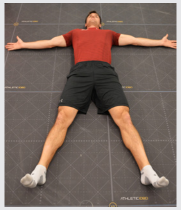

Video-Instructions:
www.swiss-ski.ch/off-snow
