# Supplementary material for: The ISPAInt Injury Prevention Programme for Youth Competitive Alpine Skiers: A Controlled 12-Month Experimental Study in a Real-World Training Setting
Source: Front Physiol. 2022 Feb 25;13:826212. doi: 10.3389/fphys.2022.826212 (PMC8929391; doi:10.3389/fphys.2022.826212)
Supplement: File C — ISPAInt programme in French. [file Data_Sheet_3.PDF]

Balgrist
Clinique universitaire

swissski

ISPA Programme de Prévention

Dynamic Bridging | Nordic Hamstring Exercise | Single Leg Squat
Dynamic Planking | Deadbug Bridging

	Statique / Dynamique	Exercice	Explications	Charge
**Ischio-jambiers 1**	Dynamique	**Dynamic Bridging**<br>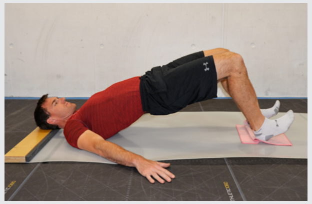<br>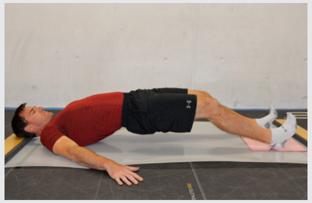	**Position de départ:**<br>• Allongé sur le dos ; tête en contact avec le sol ; pieds nus<br>• Bras écartés de 45° par rapport au corps ; paumes vers le bas<br>• Jambes pliées, position de largeur de hanche<br>• Talons sur la surface de glissement (tissu, tapis, slide, . . .)<br>• Levez le bassin jusqu'à ce que les épaules, les hanches et les genoux (vus de profil) forment une ligne<br>• Positionner les tibias à 90° par rapport aux cuisses<br>• Tout en maintenant une attitude naturelle de la colonne lombaire (lordose), tendez les muscles du tronc<br>**Déroulement de l'exercice:**<br>• Pieds en flex; éloigner les talons du corps de la position de départ (2 sec)<br>• Dès que l'extension complète du corps est atteinte, maintenir la position (1 sec)<br>• Fléchir les jambes jusqu'à ce que vous reveniez à la position de départ (2 sec)<br>• Dès que la position de départ est atteinte, changez immédiatement de direction et éloignez à nouveau les talons du corps<br>**Accent:**<br>Tout au long de l'exercice:<br>• Les épaules, les hanches et les genoux (vue de profil) forment une ligne<br>• Pas de contact au sol avec les fesses	Répétitions par série: 8–12<br>Séries: 2<br>Pause entre les séries: 1 min<br>Minutage: voir le déroulement du mouvement
**Ischio-jambiers 2**	Dynamique	**Nordic Hamstring Exercise**<br>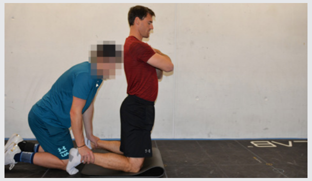<br>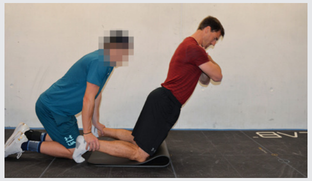<br>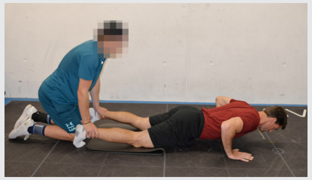	**Position de départ:**<br>• De la station à genoux sur le sol avec les pieds flex en appui (plus difficile : appui supplémentaire, p. ex. Blackroll sous les chevilles) ; pieds nus<br>• Pieds fixés aux chevilles par le partenaire<br>• Les épaules, les hanches et les genoux forment une ligne (vu de profil)<br>• Bras croisés devant la poitrine<br>**Déroulement du mouvement:**<br>• Incliner continuellement le corps vers l'avant à partir de la position de départ (3 sec)<br>• Utilisez les deux jambes pour freiner au maximum le mouvement<br>• Continuer le mouvement jusqu'à ce que la position ne puisse plus être maintenue, amortir avec les bras et revenir à la position de départ (idéalement : maintenir l'extension de la hanche)<br>**Accent:**<br>• Les épaules, les hanches et les genoux (vue de profil) forment une ligne	Répétitions par série: 3–6<br>Séries: 2<br>Pause entre les séries: 1 min<br>Minutage: voir le déroulement du mouvement<br>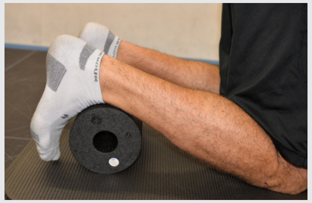

	Statique / Dynamique	Exercice	Explications	Charge
Axe des jambes 1	Dynamique	**Single Leg Squat (jambe droite)**<br>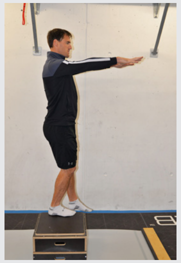 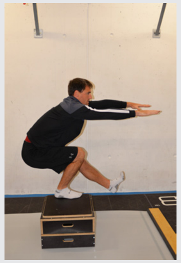	**Position de départ:**<br>• Station sur la jambe droite sur la «box» (plus difficile : à même le sol) ; pieds nus<br>• Lever les bras vers l'avant à hauteur des épaules parallèlement au sol<br>• Jambes tendues légèrement en avant<br>**Déroulement du mouvement:**<br>• Pliez la jambe d'appui jusqu'à ce que le haut de la cuisse soit parallèle au sol (2 sec)<br>• Maintenir la position (1 sec)<br>• Tendre la jambe de façon dynamique jusqu'à ce que la position de départ soit à nouveau atteinte<br>**Accent:**<br>• Contracter consciemment les muscles des fessiers<br>• L'axe de la hanche et l'axe des jambes restent stables	Répétitions par séries: 6–8<br>Séries: 2<br>Pause entre les séries: 1 min<br>Minutage: voir déroulement du mouvement<br>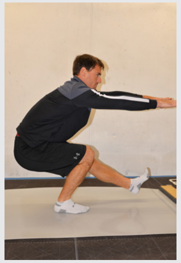
Axe des jambes 2	Dynamique	**Single Leg Squat (jambe gauche)**<br>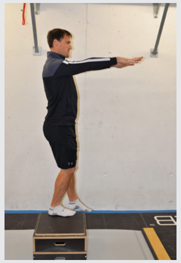 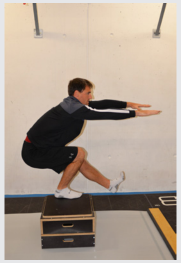	**Position de départ:**<br>• Station sur la jambe gauche sur la «box» (plus difficile : à même le sol) ; pieds nus<br>• Lever les bras vers l'avant à hauteur des épaules parallèlement au sol<br>• Jambes tendues légèrement en avant<br>**Déroulement du mouvement:**<br>• Pliez la jambe d'appui jusqu'à ce que le haut de la cuisse soit parallèle au sol (2 sec)<br>• Maintenir la position (1 sec)<br>• Tendre la jambe de façon dynamique jusqu'à ce que la position de départ soit à nouveau atteinte<br>**Accent:**<br>• Contracter consciemment les muscles des fessiers<br>• L'axe de la hanche et l'axe des jambes restent stables	Répétitions par séries: 6–8<br>Séries: 2<br>Pause entre les séries: 1 min<br>Minutage: voir déroulement du mouvement<br>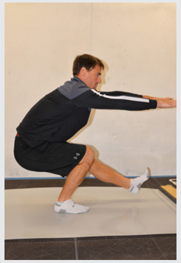

	Statique / Dynamique	Exercice	Explications	Charge
Tronc 1	Statique	**Dynamic Planking**<br>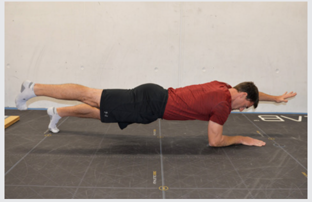	**Position de départ:**<br>• Adopter une position en appui facial, (sur les avant-bras et les orteils) ; pieds nus<br>• Coudes directement sous l'épaule ; avant-bras alignés parallèlement à l'axe longitudinal du corps<br>• La tête, les épaules, les hanches et les genoux forment une ligne (vu de profil)<br>• Les muscles du torse et les muscles fessiers sont contractés ; légère tension dans la région des omoplates<br>**Déroulement du mouvement:**<br>• Élévation opposée des bras et des jambes tendus<br>• Maintenir la position à chaque fois pendant 2 secondes<br>**Accent:**<br>• Le torse et les hanches restent stables (tête, épaules, hanches et genoux forment une seule ligne)<br>• Maintenir une attitude naturelle de la colonne lombaire (lordose)	Répétitions par série: 20–30<br>Séries: 2<br>Pause entre les séries: 1 min<br>Minutage: voir déroulement du mouvement
Tronc 2	Statique	**Deadbug Bridging**<br>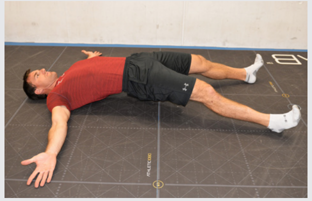<br>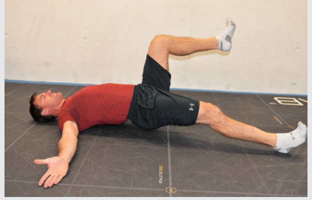	**Position de départ:**<br>• Allongé sur le dos ; bras à 90° par rapport au corps ; paumes vers le haut ; pieds nus<br>• Jambes tendues et écartées (talons et coudes à peu près à la même distance du centre du corps), pointe des pieds effacée<br>• Métaphore : «Tirer le nombril vers le menton»<br>• Augmenter la tension dans le torse<br>**Déroulement du mouvement:**<br>• Soulevez légèrement le bassin et tenez-le au-dessus du sol à une hauteur d'un poing<br>• Tirer les genoux alternativement vers la poitrine jusqu'à ce que la cuisse soit perpendiculaire au sol (2 sec)<br>• Maintenir la position (3 sec)<br>• Descendre la jambe lentement (2 secondes) et poser à nouveau le pied de façon contrôlée<br>**Accent:**<br>• Le torse et les hanches restent stables<br>• Maintenir la position de départ de la colonne vertébrale («tirer le nombril vers le menton»)<br>• La tête reste au sol	Répétition par série: 4–6<br>Séries: 2<br>Pause entre les séries: 30 Sek<br>Minutage : voir déroulement du mouvement<br>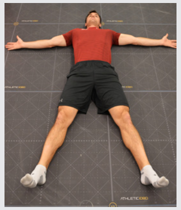

Video-Instructions:
www.swiss-ski.ch/fr/off-snow
