# Supplementary material for: The ISPAInt Injury Prevention Programme for Youth Competitive Alpine Skiers: A Controlled 12-Month Experimental Study in a Real-World Training Setting
Source: Front Physiol. 2022 Feb 25;13:826212. doi: 10.3389/fphys.2022.826212 (PMC8929391; doi:10.3389/fphys.2022.826212)
Supplement: File D — ISPAInt programme in Italian. [file Data_Sheet_4.PDF]

Balgrist
Clinica universitaria

swissski

ISPA Programma di prevenzione

Dynamic Bridging | Nordic Hamstring Exercise | Single Leg Squat
Dynamic Planking | Deadbug Bridging

	Statico / dinamico	Esercizio	Spiegazione	Compito
**Ischiocrurali 1**	Dinamico	**Dynamic Bridging**<br>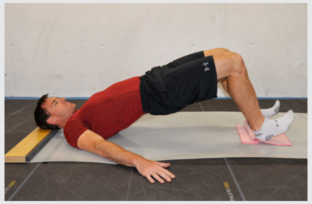<br>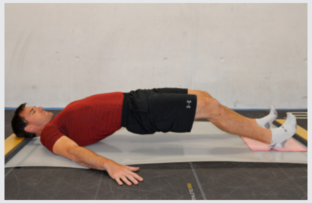	**Posizione di partenza:**<br>• Supini, testa appoggiata a terra, piedi nudi<br>• Braccia a 45° dal corpo con i palmi delle mani verso il basso<br>• Gambe flesse e divaricate alla stessa larghezza delle anche<br>• Talloni appoggiati alla superficie di scorrimento (telo, tappeto, piano scorrevole)<br>• Sollevare il bacino finché spalle, anche e ginocchia non risultino allineate (viste di profilo)<br>• Posizionare le tibie in un angolo di 90° rispetto alle cosce<br>• Tendere i muscoli del tronco, mantenendo una postura naturale del tratto lombare (lordosi)<br><br>**Svolgimento dell'esercizio:**<br>• Con i piedi a martello allontanare i talloni dal corpo dalla posizione di partenza (2 s)<br>• Una volta raggiunta la completa estensione del corpo, mantenere la posizione (1 s)<br>• Flettere le gambe fino a tornare alla posizione di partenza (2 sec.)<br>• Una volta riacquistata la posizione di partenza, cambiare immediatamente direzione e tornare ad allontanare i talloni dal corpo<br><br>**Focus:**<br>Durante tutto l'esercizio:<br>• spalle, anche e ginocchia rimangono allineate (viste di profilo)<br>• i glutei non toccano terra	Ripetizioni per serie: 8–12<br>Serie: 2<br>Recupero tra le serie: 1 min.<br>Tempi: si veda lo svolgimento del movimento
**Ischiocrurali 2**	Dinamico	**Nordic Hamstring Exercise**<br>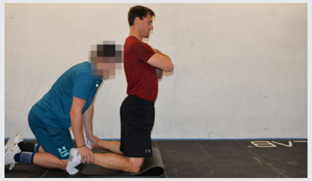<br>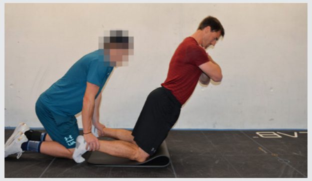<br>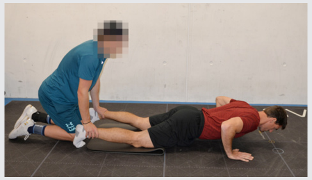	**Posizione di partenza:**<br>• In ginocchio a terra con i piedi nudi a martello appoggiati (versione più complessa: supporto aggiuntivo per es. Blackroll sotto le caviglie)<br>• Un partner afferra le caviglie per mantenere i piedi a terra<br>• Spalle, anche e ginocchia allineate (viste di profilo)<br>• Braccia incrociate sul petto<br><br>**Svolgimento del movimento:**<br>• Inclinare progressivamente il corpo in avanti dalla posizione di partenza (3 sec.)<br>• Far forza sulle gambe per rallentare al massimo il movimento<br>• Continuare a inclinarsi fintanto che sia possibile mantenere la posizione, quindi ammortizzare la caduta con le braccia e tornare alla posizione di partenza (l'ideale è mantenere l'estensione dell'anca)<br><br>**Focus**:<br>• spalle, anche e ginocchia rimangono allineate (viste di profilo)	Ripetizioni per serie: 3–6<br>Serie: 2<br>Recupero tra le serie: 1 min.<br>Tempi: si veda lo svolgimento del movimento<br>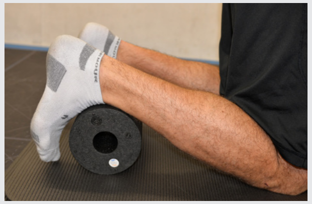

	Statico / dinamico	Esercizio	Spiegazione	Compito
**Asse delle gambe 1**	Dinamico	**Single Leg Squat (gamba destra)**<br>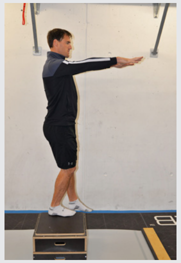 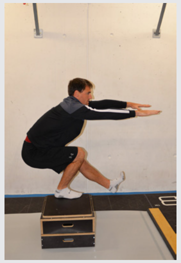	**Posizione di partenza:**<br>• In piedi sul «box» poggiando sulla gamba destra (più complesso: direttamente a terra), piedi nudi<br>• Sollevare le braccia in avanti, portandole all'altezza delle spalle, parallele a terra<br>• Gambe tese leggermente in avanti<br>**Svolgimento del movimento:**<br>• Flettere la gamba d'appoggio fintanto che la parte alta della coscia non risulti parallela a terra (2 sec.)<br>• Mantenere la posizione (1 sec.)<br>• Distendere la gamba in modo dinamico fino a raggiungere nuovamente la posizione di partenza<br>**Focus:**<br>• Contrarre consapevolmente i muscoli dei glutei<br>• Mantenere stabilmente in asse l'anca e le gambe	Ripetizioni per serie: 6–8<br>Serie: 2<br>Recupero tra le serie: 1 min.<br>Tempi: si veda lo svolgimento del movimento<br>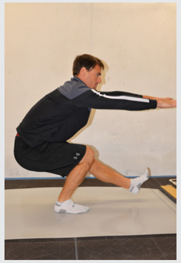
**Asse delle gambe 2**	Dinamico	**Single Leg Squat (gamba sinistra)**<br>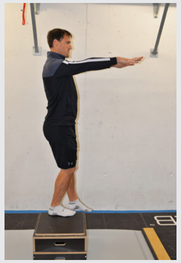 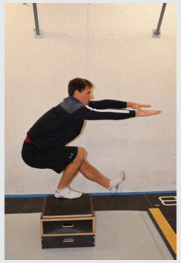	**Posizione di partenza:**<br>• In piedi sul «box» poggiando sulla gamba sinistra (più complesso: direttamente a terra), piedi nudi<br>• Sollevare le braccia in avanti, portandole all'altezza delle spalle, parallele a terra<br>• Gambe tese leggermente in avanti<br>**Svolgimento del movimento:**<br>• Flettere la gamba d'appoggio fintanto che la parte alta della coscia non risulti parallela a terra (2 sec.)<br>• Mantenere la posizione (1 sec.)<br>• Distendere la gamba in modo dinamico fino a raggiungere nuovamente la posizione di partenza<br>**Focus:**<br>• Contrarre consapevolmente i muscoli dei glutei<br>• Mantenere stabilmente in asse l'anca e le gambe	Ripetizioni per serie: 6–8<br>Serie: 2<br>Recupero tra le serie: 1 min.<br>Tempi: si veda lo svolgimento del movimento<br>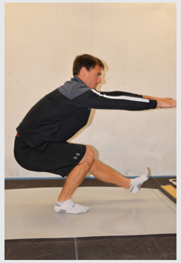

	Statico / dinamico	Esercizio	Spiegazione	Compito
Tronco 1	Statico	**Dynamic Planking**<br>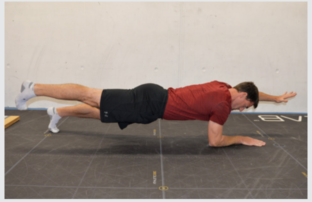	**Posizione di partenza:**<br>• Proni in appoggio (sugli avambracci e puntando gli alluci), sguardo verso terra e piedi nudi<br>• Gomiti direttamente sotto le spalle, avambracci allineati e paralleli all'asse longitudinale del corpo<br>• Testa, spalle, anche e ginocchia allineate (viste di profilo)<br>• I muscoli del torso e quelli dei glutei sono contratti con una lieve tensione nell'area scapolare<br>**Svolgimento del movimento:**<br>• Sollevare contemporaneamente braccio e gamba opposti, entrambi tesi<br>• Mantenere la posizione per 2 secondi a ogni sollevamento<br>**Focus:**<br>• Torso e anche stabili in posizione (testa, spalle, anche e ginocchia allineate)<br>• Mantenere una postura naturale del tratto lombare (lordosi)	Ripetizioni per serie: 20–30<br>Serie: 2<br>Recupero tra le serie: 1 min.<br>Tempi: si veda lo svolgimento del movimento
Tronco 2	Statico	**Deadbug Bridging**<br>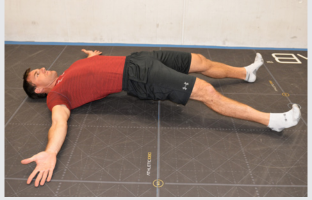<br>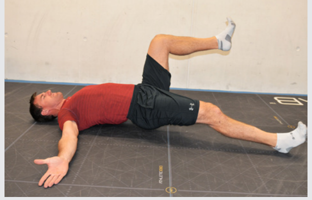	**Posizione di partenza:**<br>• Supini, braccia a 90° rispetto al corpo con i palmi delle mani verso l'alto, piedi nudi<br>• Gambe tese e divaricate (talloni e gomiti circa alla stessa distanza dal centro del corpo), punte dei piedi rivolte all'esterno<br>• Immagine: «portare l'ombelico verso il mento»<br>• Aumentare la tensione del torso<br>**Svolgimento del movimento:**<br>• Sollevare leggermente il bacino e mantenerlo a una distanza da terra pari a un pugno<br>• Flettere le ginocchia verso il petto, alternando le gambe, fintanto che le cosce non risultino perpendicolari a terra (2 sec.)<br>• Mantenere la posizione (3 sec.)<br>• Far scendere la gamba lentamente (2 secondi) e appoggiare nuovamente il piede a terra, mantenendo il controllo<br>**Focus:**<br>• Torso e anche stabili in posizione<br>• Mantenere la posizione di partenza della colonna vertebrale («portare l'ombelico verso il mento»)<br>• La testa deve rimanere a terra	Ripetizioni per serie: 4–6<br>Serie: 2<br>Recupero tra le serie: 30 sec.<br>Tempi: si veda lo svolgimento del movimento<br>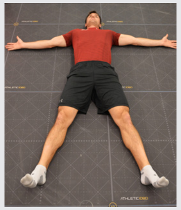

Video-Instructions:
www.swiss-ski.ch/off-snow
